# Investigation of human iPSC-derived cardiac myocyte functional maturation by single cell traction force microscopy

**DOI:** 10.1371/journal.pone.0194909

**Published:** 2018-04-04

**Authors:** Matthew Wheelwright, Zaw Win, Jennifer L. Mikkila, Kamilah Y. Amen, Patrick W. Alford, Joseph M. Metzger

**Affiliations:** 1 Department of Integrative Biology and Physiology, University of Minnesota Medical School, Minneapolis, Minnesota, United States of America; 2 Department of Biomedical Engineering, University of Minnesota, Minneapolis, Minnesota, United States of America; University of Tampere, FINLAND

## Abstract

Recent advances have made it possible to readily derive cardiac myocytes from human induced pluripotent stem cells (hiPSC-CMs). HiPSC-CMs represent a valuable new experimental model for studying human cardiac muscle physiology and disease. Many laboratories have devoted substantial effort to examining the functional properties of isolated hiPSC-CMs, but to date, force production has not been adequately characterized. Here, we utilized traction force microscopy (TFM) with micro-patterning cell printing to investigate the maximum force production of isolated single hiPSC-CMs under varied culture and assay conditions. We examined the role of length of differentiation in culture and the effects of varied extracellular calcium concentration in the culture media on the maturation of hiPSC-CMs. Results show that hiPSC-CMs developing in culture for two weeks produced significantly less force than cells cultured from one to three months, with hiPSC-CMs cultured for three months resembling the cell morphology and function of neonatal rat ventricular myocytes in terms of size, dimensions, and force production. Furthermore, hiPSC-CMs cultured long term in conditions of physiologic calcium concentrations were larger and produced more force than hiPSC-CMs cultured in standard media with sub-physiological calcium. We also examined relationships between cell morphology, substrate stiffness and force production. Results showed a significant relationship between cell area and force. Implementing directed modifications of substrate stiffness, by varying stiffness from embryonic-like to adult myocardium-like, hiPSC-CMs produced maximal forces on substrates with a lower modulus and significantly less force when assayed on increasingly stiff adult myocardium-like substrates. Calculated strain energy measurements paralleled these findings. Collectively, these findings further establish single cell TFM as a valuable approach to illuminate the quantitative physiological maturation of force in hiPSC-CMs.

## Introduction

Over the past several years it has become possible to efficiently derive robust, spontaneously contracting cardiac myocytes (hiPSC-CMs) from human induced pluripotent stem cells (hiPSCs)[1, 2, 3]. Regarded as a viable source of virtually unlimited human cardiac muscle tissue, researchers and clinicians have begun utilizing hiPSC-CMs as potential source for therapeutic cell-based repair via transplantation into host[4] and for cellular and tissue models of cardiac disease[5, 6]. Since their discovery, considerable efforts have been underway to assess and quantify hiPSC-CM contractile function and to address the developmental state and physiological maturity of the hiPSC-CM[7, 8, 9, 10].

The central measure of the physiologic function of a cardiac myocyte, and the essential purpose of the cell, is force production. To date, several groups have implemented assays to measure force production of hiPSC-CMs, either as a syncytium[11, 12] or population of cells on a thin film[13], or as single cells using micropost arrays[14] or, most recently, by using traction force microscopy[15]. Force production is an important quantitative index of cardiac myocyte maturity. It has been shown that human fetal cardiac myofibrils produce less force than adult cardiac myofibrils, and that this increases over time in human development[16]. Furthermore, isometric force in skinned myocytes from mice and sheep increase as gestational age increases[17, 18]. Quantitative assessment of hiPSC-CMs during culture is, therefore, important to ascertain as a key functional benchmark for maturation. In this study, we examine the important question of whether increased age (length of differentiation) of hiPSC-CMs can improve maturation.

We further hypothesized that hiPSC culture media composition is another critical factor in guiding hiPSC-CM functional (force) maturation. It is well known that the physiologic extracellular calcium concentration in mammalian interstitial spaces is between 1.5–2.0 mM[19], providing a strong electrochemical gradient opposite a much smaller intracellular calcium concentration in heart muscle[20, 21]. However, the calcium concentration in RPMI, which is the basal media used in differentiation and growth of hiPSC-CMs used in several well-cited protocols[2, 3], is sub-physiological at 0.42 mM[22]. Numerous groups have shown that extracellular calcium and calcium signaling play a significant role in cardiac development, especially in cardiac myocyte hypertrophy[23, 24]. With this information, we hypothesized that a physiological extracellular calcium concentration in the culture media is necessary to further promote maturation of force production in hiPSC-CMs.

Intimately related to the myocyte’s ability to produce force is its morphology, including total cell area, length and width. The use of micropattern printing to design and manipulate the shape of neonatal ventricular cardiac myocytes shows a range of aspect ratios that result in maximal force production, presumably by improved sarcomere and myofibril alignment[25, 26]. Recent studies in hiPSC-CMs demonstrate increased force in longer cells compared to shorter ones[27]. Investigations of the relationship between cell size and force is important, as cardiac myocyte size changes dramatically during cardiac development with the transition from immature to mature cardiac myocyte, involving a significant increase in cell area[28].

Force production in both adult and fetal cardiac myocytes is known to be highly dependent upon the load against which the cell is contracting. This includes the stiffness of the immediate microenvironment[29]. Alterations in the myocyte microenvironment can have significant effects in overall heart performance. For example, cardiac output is severely compromised in cases where tissue stiffness changes drastically, as in fibrotic diseases of the heart[30]. The stiffness of the human heart also changes during development in utero; however, the elastic modulus of the developing myocardium is debated and varies markedly depending on the method of measurement, as does measurement of the stiffness of other tissues[31]. To date, most studies report that the elastic modulus of the myocardium increases with age[32, 33]. Furthermore, the stiffness of myocardium is well known to be an important regulator of cardiac myocyte function [34, 35, 36]. Thus, we hypothesized that the ability to produce force against varying levels of stiffness is an important marker of human heart muscle physiologic maturity.

Therefore, in the present study, we investigated the functional maturation status of hiPSC-CMs using single cell microprinting and traction force microscopy to measure force production of isolated myocytes. We examined force in the context of several physiologically relevant environmental parameters: first, we tested hiPSC-CMs contractile maturation by comparing them to neonatal rat ventricular myocytes (NRVMs), with a focus on cell morphology and geometry. Then, we measured force in response to substrates of varying stiffness. Finally, we tested hiPSC-CM development after being cultured in varied physiologic extracellular calcium conditions.

## Results

### HiPSC-CMs align and contract along a single axis

**Fig 1:**
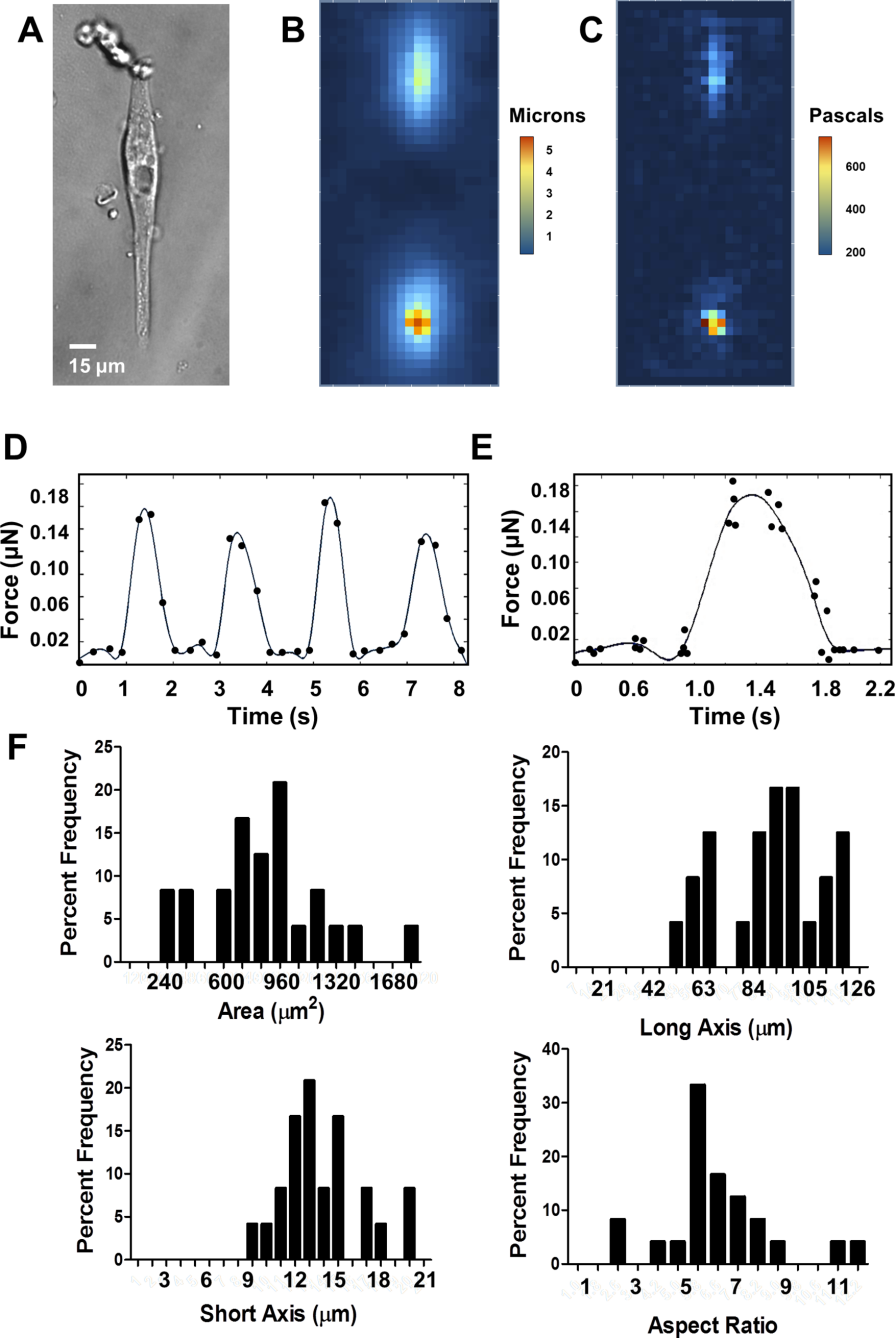
HiPSC-CMs and measurement of force by TFM. A. Representative cell, 30 days post-differentiation on a 9.8 kPa substrate. B. Heat map showing magnitudes of deformation strain of the substrate under the representative cell. C. Heat map showing magnitudes of stress of the representative cell calculated from strain of the substrate. D. Total force of a single cell over time with respect to baseline at the point t = 0 seconds, over four contractions paced at 0.5 Hz, fitted with a smoothed spline curve. E. Total force of a single cell over time, average of four contractions, fitted with a smoothed spline curve. F. Histograms showing distribution of cell geometries.

Human iPSC-CMs were transferred to polyacrylamide (PAA) gels that had been micropatterned with laminin rectangles with an area of 2000 μm^2^, which is larger than the expected area based on previous measurements of hiPSC-CMs[6], and an aspect ratio of 7:1, which has been reported as an ideal aspect ratio for NRVM force production[19]. This allowed individual hiPSC-CMs to adhere to the substrate and occupy an area of up to 2000 μm^2^, allowing them to take on their preferred size and geometry. Here, hiPSC-CMs formed an elongated geometry aligned along the direction of the long axis of the patterned area (Fig 1A). Most hiPSC-CMs formed geometries with an area smaller than 2000 μm^2^and an aspect ratio slightly smaller than 7:1 (Fig 1F).

Although hiPSC-CMs grown in 2-dimensional culture beat synchronously, isolated single cells are heterogeneous and frequently do not beat at the same rate. Thus, patterned single hiPSC-CMs were paced via field stimulator at 0.5 Hz and 35 mV. Pacing at 0.5 Hz, a rate frequently used in experiments on isolated adult myocytes[37], allowed the cells to completely return to baseline between contractions, which is important in defining the parameters of contraction. Paced myocytes contracted along their long axes, creating visible deformations in the fluorescent bead-containing substrate (S1 Movie). Particle image velocimetry analysis showed greatest substrate displacement towards the ends of the hiPSC-CMs, as well as in areas surrounding the ends of the cells (Fig 1B). Traction force analysis showed that the largest traction stress was developed in the substrate at these same locations (Fig 1C).

Averaged contractions fitted with a smoothed spline curve showed a force development curve resembling that of adult cardiac myocytes (Fig 1D and 1E)[37, 38]. HiPSC-CM calculated maximum force measurements were on the order of 10^−8^ N, which is in line with measurements previously reported by others[21]. The high resolution of the fluorescent images necessary for accurate quantification of traction vectors allowed us to accurately capture peak and return to baseline position of contracting hiPSC-CMs, as seen in Fig 1D. To further validate our measurements, force trace ensembles of four contractions demonstrated a well-defined and well-resolved contractile cycle (Fig 1E) consistent with single trace data (Fig 1D).

### Heterogeneous cell geometry affects contractility

**Fig 2:**
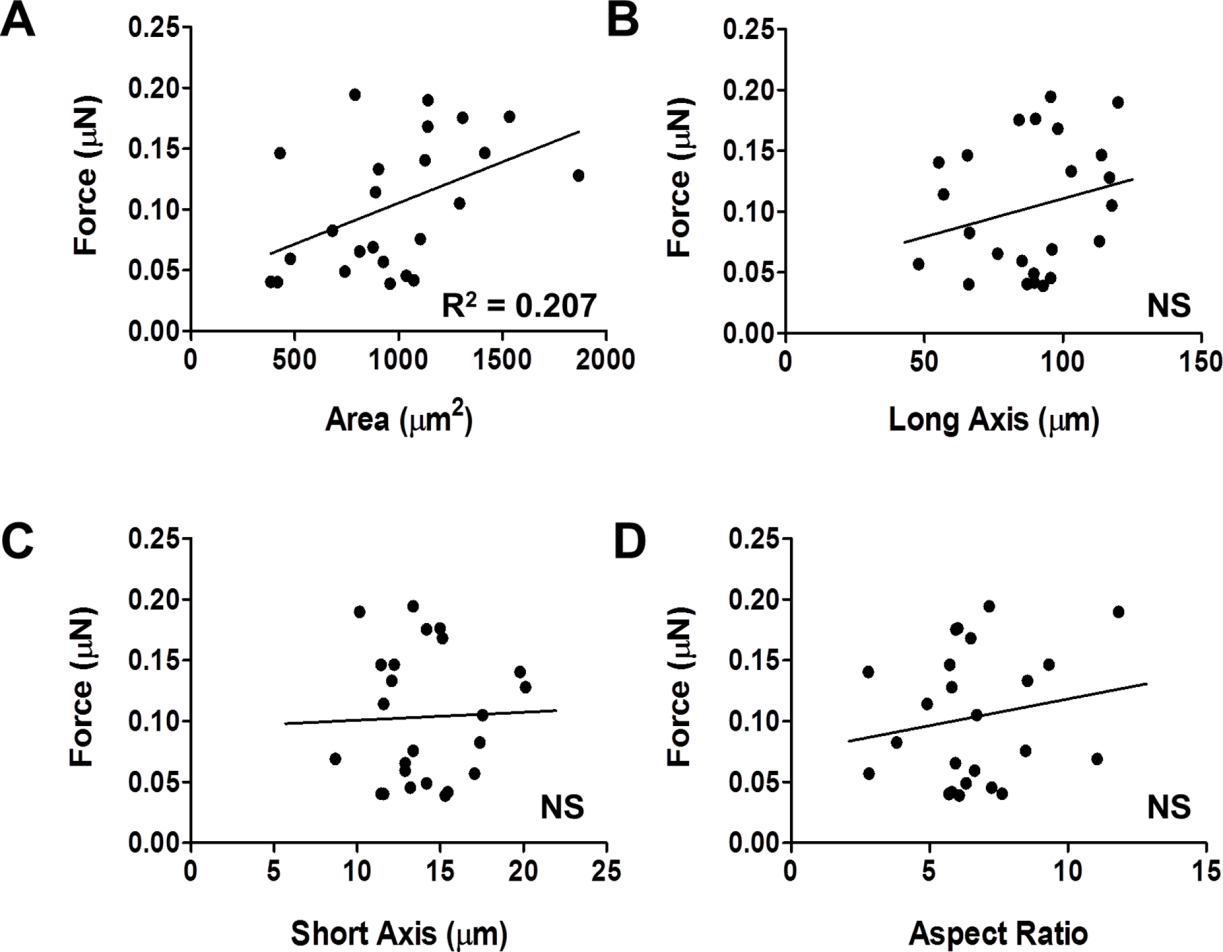
Effects of single cell d90 hiPSC-CM morphology on force production. A. Peak force versus cell area, R^2^ = 0.21, P<0.03, n = 24. B. Peak force versus long axis (axis of contraction). C. Peak force versus short axis (perpendicular to axis of contraction). D. Peak force versus aspect ratio (long axis/short axis).

Differentiation protocols can result in the development of a heterogeneous population of cardiac myocytes, as evidenced by varied electrophysiological parameters[39], calcium handling[40] and gene expression profiles[40]. To address this, we sought to examine the potential effects of geometric heterogeneity on physiologic force production. Micropatterned PAA constructs were designed as a rectangle with a 7:1 aspect ratio and a 2000 μm^2^ surface area. HiPSC-CMs that have been cultured on the constructs are able to occupy an area of up to 2000 μm^2^, in their preferred aspect ratio, which ranged from 4:1 to as long as 10:1, with a mean of 6.6:1 (Fig 1F). Based on this outcome, we examined the effects of cell geometry on contractility. We first measured total force production of d90 hiPSC-CMs on a 9.8 kPa substrate. There was a significant positive correlation between cell size and total force produced (Fig 2A, R^2^ = 0.21, P = 0.02). However, we found no correlation between long axis (length), short axis (width), or aspect ratio and force (Fig 2B–2D). Based on these findings, for the remainder of this paper we report total force, as well as force per unit area, which we refer to here as normalized force.

### Development of hiPSC-CMs under prolonged culture conditions

It is widely accepted that hiPSC-CMs phenotypically resemble immature cardiac myocytes and, depending on the studied characteristics, resemble embryonic cardiac myocytes[41], fetal cardiac myocytes[8, 16] or neonatal cardiac myocytes[13]. Furthermore, it has been hypothesized that with increased culture time, a more mature phenotype can be obtained. Accordingly, we examined TFM-based force production of hiPSC-CMs after 14 days, 30 days, or 90 days in culture and compared to NRVMs.

**Fig 3:**
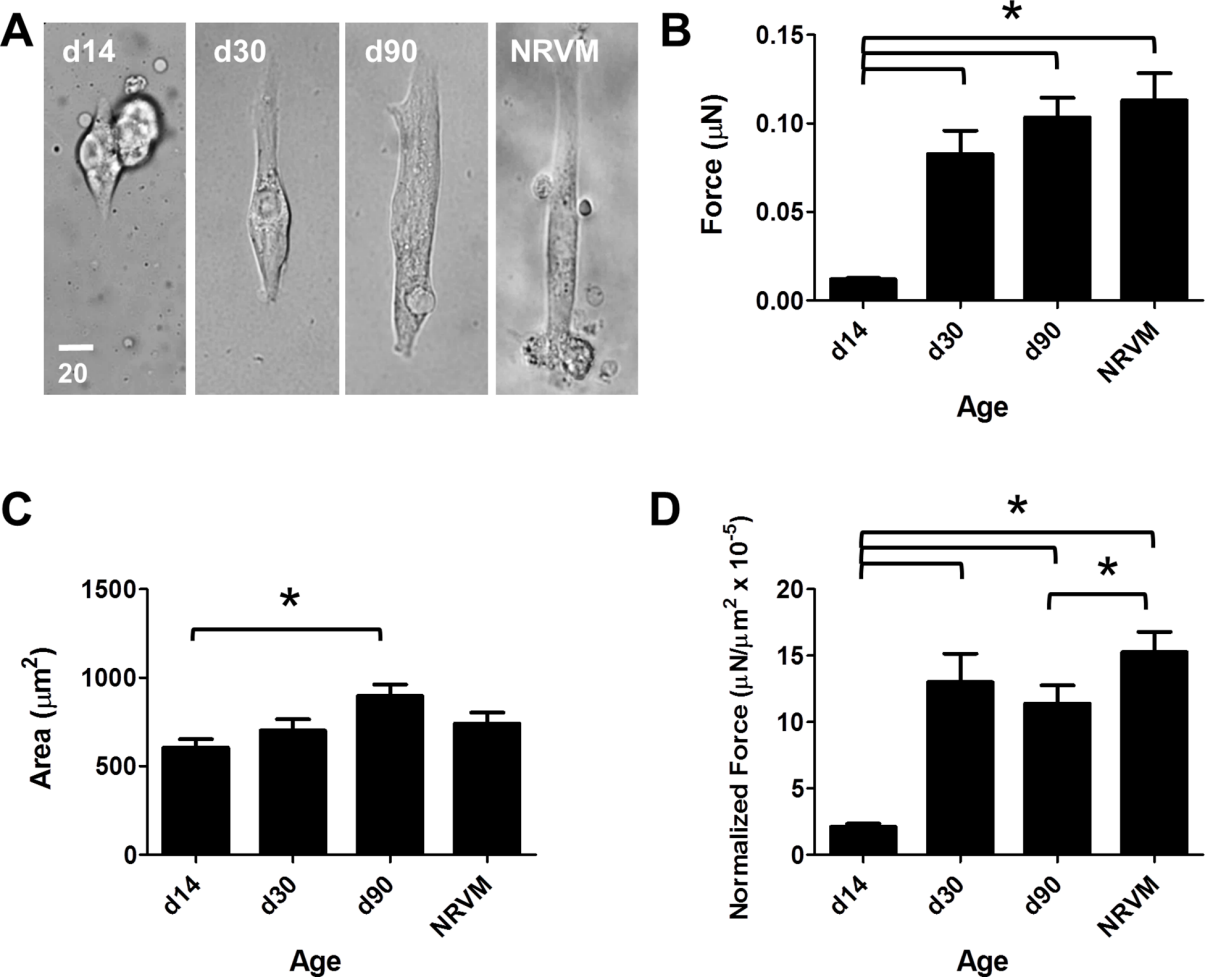
Effects of length of hiPSC differentiation on force production. Data shown as mean ± SEM. A. Representative hiPSC-CMs from day 14, day 30, and day 90 post-differentiation, and representative NRVM on substrate with modulus 9.8 kPa. B. Total force versus length of differentiation (mean = 0.012 ± 0.001 μN, n = 17; 0.083 ± 0.013 μN, n = 15; 0.103 ± 0.011 μN, n = 24; 0.113 ± 0.016 μN, n = 12). C. Cell area versus length of differentiation (mean = 605.7 ± 47.1 μm^2^, 702.3 ± 63.2 μm^2^, 898.2 ± 64.4 μm^2^, 741.9 ± 61.4 μm^2^). D. Normalized force versus length of differentiation in culture (mean = 2.12 ± 0.23 mN/mm2 x 10^−5^, 13.0 ± 2.14 mN/mm2 x 10^−5^, 11.4 ± 1.4 mN/mm2 x 10^−5^, 15.3 ± 1.5 mN/mm2 x 10^−5^).

At d14, hiPSC-CMs produce small but detectable amounts of force, whereas d30 cells produced significantly more total force and normalized force (one-way ANOVA P < 0.0001) (Fig 3A–3C). At d90, single hiPSC-CMs produced more total force than at d30. However, as they were also larger, normalized force was not significantly different (Fig 3D). However, d90 cells produced significantly more total force (P < 0.0001) and normalized force (P < 0.0001) than d14 cells. D90 cells were significantly larger than d14 cells (P = 0.01). NRVMs showed similar cell size and total force produced compared to d90 hiPSC-CMs; however, they had significantly higher normalized force (P < 0.05).

### Effects of substrate mechanics on contractility

**Fig 4:**
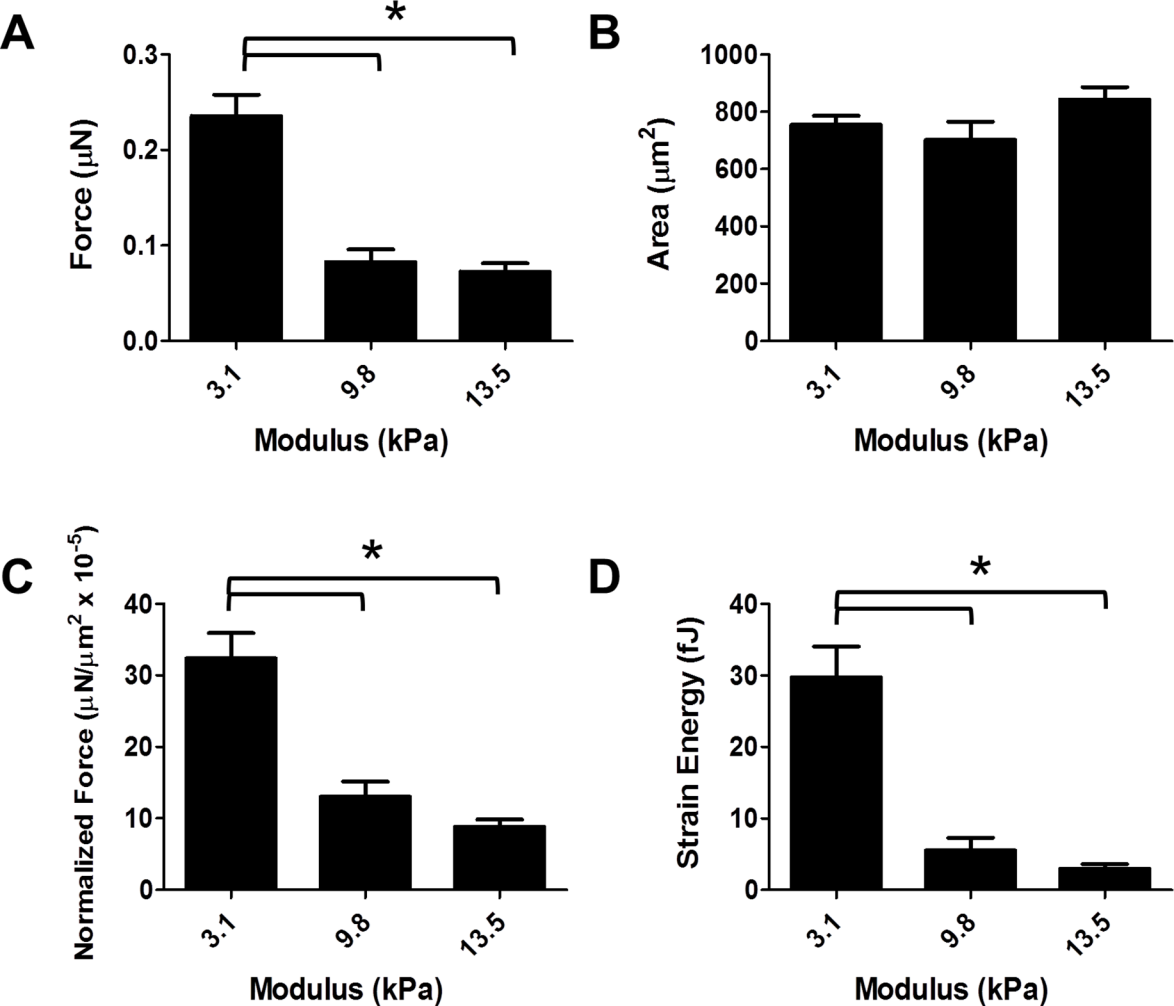
Effects of substrate stiffness on d30 hiPSC-CM force production. Data shown as mean ± SEM. A. Total force versus substrate elastic modulus (mean = 0.236 ± 0.02 μN, n = 21; 0.083 ± 0.01 μN, n = 15; 0.075 ± 0.01 μN, n = 22). B. Area versus substrate elastic modulus (mean = 754.9 ± 31.5 μm^2^, 702.3 ± 63.2 μm^2^, 851.6 ± 43.7 μm^2^). C. Normalized force versus substrate elastic modulus (mean = 32.4 ± 3.5 mN/mm2 x 10^−5^, 13.0 ± 2.1 mN/mm2 x 10^−5^, 9.1 ± 1.0 mN/mm2 x 10^−5^). D. Strain energy versus substrate elastic modulus (mean = 29.7 ± 4.3 fJ, 5.5 ± 1.7 fJ, 3.1 ± 0.6 fJ).

Cardiac myocyte work adapts significantly during development[42]. Furthermore, cardiac myocytes produce different amounts of traction force in response to altered mechanical environments[15, 43]. To investigate whether this holds true for hiPSC-CMs, we cultured hiPSC-CMs for 30 days under standard growth conditions (see Methods), then transferred isolated hiPSC-CMs to PAA gels with a defined modulus of 3.1, 9.8, or 13.5 kPa. HiPSC-CMs had decreased total force (One-way ANOVA, P < 0.0001) (Fig 4A) and normalized force (One-way ANOVA P < 0.0001) (Fig 4C) as a function of increased substrate stiffness. HiPSC-CMs on the 3.1 kPa modulus substrate produced significantly more force than on 9.8 or 13.5 kPa (Fig 4A and 4C).

Cell area was not significantly different between conditions, indicating the range of substrate stiffness tested was not sufficient to induce changes in cell spreading, and that cell spreading was not the cause of differential force production (Fig 4B). At a substrate modulus higher than 13.5 kPa, bead displacement was very small, resulting in a poor signal-to-noise ratio (data not shown). We also calculated strain energy generated by each cell during a full contraction and found a decrease in strain energy with increasing stiffness. This correlated to decreased force production, with cells on a substrate with a modulus of 3.1 kPa substrates generating significantly more energy than those on substrates with a modulus of 9.8 or 13.5 kPa substrates (One-way ANOVA P < 0.0001).

### Effects of culture media extracellular calcium content on hiPSC-CM development

**Fig 5:**
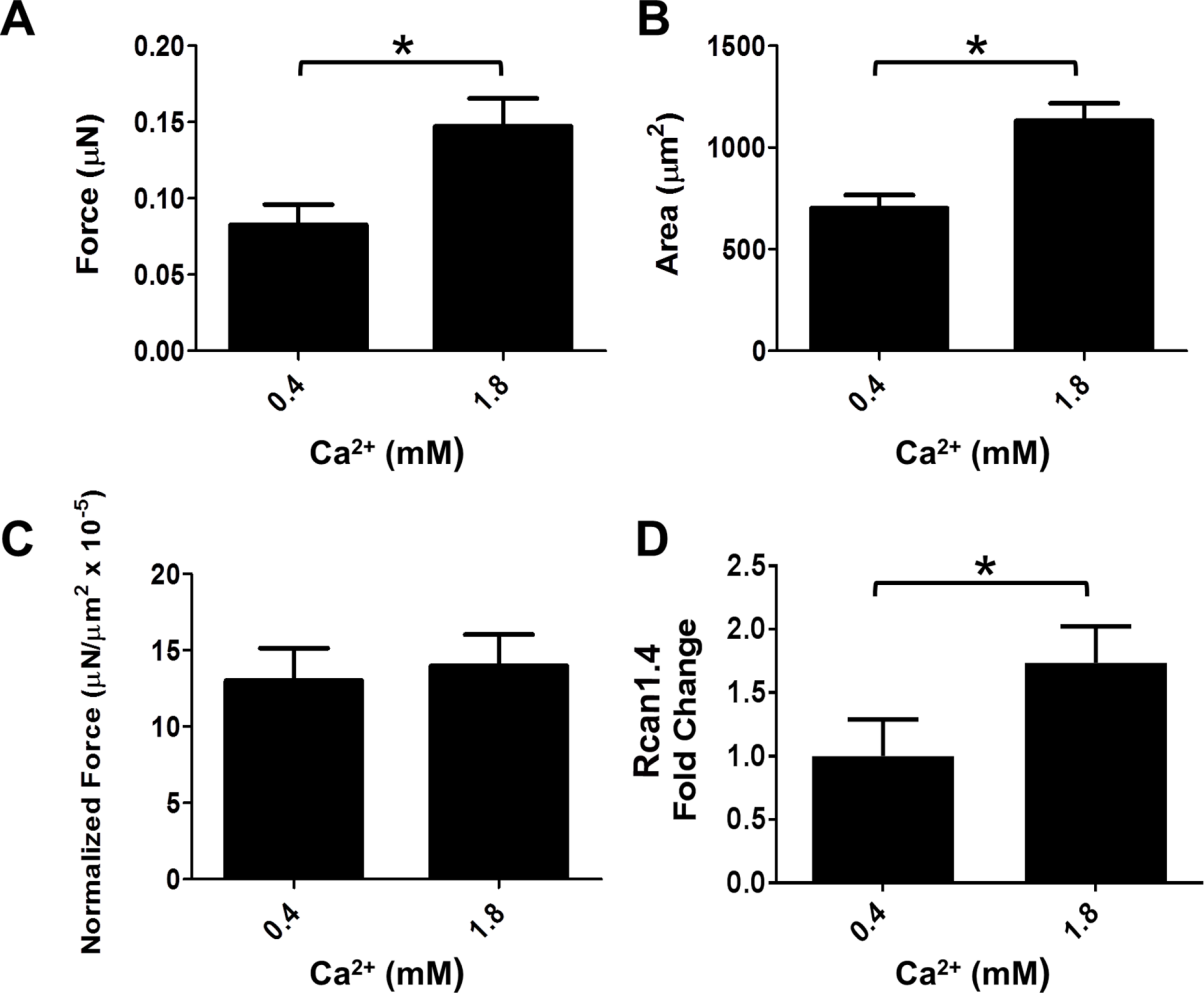
Effects of calcium concentration in growth media on d30 hiPSC-CM force production on a substrate with modulus 9.8 kPa. Data shown as mean ± SEM. A. Total force versus calcium concentration (mean = 0.083 ± 0.013 μN, n = 15; 0.147 ± 0.02 μN, n = 14). B. Cell area versus calcium concentration (mean = 702.3 ± 63.2 μm^2^, 1130.3 ± 86.2 μm^2^). C. Normalized force versus calcium concentration (mean = 13.0 ± 2.14 mN/mm2 x 10^−5^, 14.0 ± 2.0 mN/mm2 x 10^−5^). D. Rcan1.4 mRNA fold change (low Ca^2+^ group mean = 1.00 ± 0.29, n = 8; physiological Ca^2+^ mean, 1.74 ± 0.29, n = 7)(P = 0.02).

From well-cited protocols[12], the hiPSC-CM growth media contains sub-physiological levels of calcium (~0.42 mM), whereas physiologic extracellular calcium concentrations are much higher, ranging from 1.3 mM[44] to 2.0 mM[37, 45, 46]. To examine the effects of long term media calcium concentrations on hiPSC-CM function, we cultured hiPSC-CMs in growth media that had been supplemented with CaCl up to 1.8 mM Ca^2+^, beginning on the day that they began spontaneously contracting (d7), and continuously until they were tested (d30). They were all assayed in standard growth medium (Ca^2+^ concentration 0.4 mM). HiPSC-CMs developing in media with physiologic calcium levels produced greater total force than cells grown in standard growth medium, when assayed in standard growth medium (Ca^2+^ concentration 0.4 mM) on a 9.8 kPa substrate (P = 0.0073) (Fig 5A). Additionally, these hiPSC-CMs were significantly larger (P = 0.0004) (Fig 5B). However, normalized force was not significantly different between the two groups (p = 0.75) (Fig 5C).

In order to examine the mechanism whereby the increased extracellular calcium levels in the culture media contribute to altered morphology and contractility, we measured calcineurin/NFAT effector activity by quantifying Rcan1.4 mRNA, which is directly influenced by the calcineurin/NFAT pathway[47]. We found a significant 1.74-fold increase in Rcan1.4 in d30 hiPSC-CMs that had been cultured in 1.8 mM Ca^2+^ containing media compared to the standard 0.4 mM Ca^2+^ media composition (Fig 5D, P = 0.02).

## Discussion

Human induced pluripotent stem cell-derived cardiac myocytes are an attractive model system for experimental therapeutic discovery and as a potential cell/tissue source for regenerative therapy in diseased hearts. However, a significant obstacle to realizing this potential is the physiologic immaturity of hiPSC-CMs relative to adult cardiac myocytes. In this study, we utilized single cell micropatterning with traction force microscopy for quantitative assessment and investigation of absolute force production in isolated single hiPSC-CMs. Relative to other methods of assaying cardiac myocyte contractility, single-cell traction force microscopy eliminates potential confounding effects of neighboring cells, including myocytes and fibroblasts. We report single cell hiPSC-CM peak contractile force in a range of 10^−10^ to 10^−9^ N, which is comparable to other groups[27], although lower than some other studies[15]. This is likely due to the regularization factor we used in the stress calculation, which has been shown to reduce noise, but can also result in lower calculated overall stress values[48, 49].

Our study has several main new findings. Using microfabrication techniques to guide hiPSC-CM area and aspect ratio, in adopting a rectangular shape with a single contractile axis force vector, shows that force production correlates with overall cell area but not length, width, or aspect ratio. This led us to normalize all force measurements to cell area in order to obtain a more accurate representation of cellular contractile performance, termed here as normalized force, which is a different value than calculated stress. While aspect ratio did not significantly correlate here to total force, aspect ratios were distributed normally around with a mean between 6:1–7:1, which is very close to the aspect ratio that other groups have determined for hiPSC-CMs and NRVMs to produce maximal force[25, 27] These results, although initially appearing contradictory to other published studies[21], suggest that when hiPSC-CMs are allowed to adopt a physiologically relevant geometry, they prefer to assume an optimal aspect ratio for maximal force production in that individual cell.

Our data further demonstrates that hiPSC-CMs progress in terms of force output similarly to the natural embryonic development of cardiac myocytes, wherein normalized force increases as myocytes mature in vivo[16, 17]. Results show that hiPSC-CM normalized force increases as a function of time in culture, which is in line with results from other research groups[15]. Analysis of single hiPSC-CMs, compared to neonatal rat ventricular myocytes under identical assay conditions, show that hiPSC-CMs produce comparable total force as NRVMs. These results corroborate previous findings from our lab that hiPSC-CMs cultured for prolonged periods of time begin to express some adult specific isoforms of contractile proteins, such as cTnI, although still reflecting an immature expression profile compared to the adult myocardium[9]. While instructive, these results also show that it is not yet been possible to achieve physiologic maturation in terms of force output approaching that of adult cardiac myocytes.

We discovered that ionic content of media for long-term hiPSC-CM culture has important outcomes in terms of cellular maturation and force output. The effects of varied extracellular calcium on the developing heart has been studied recently[25, 26], with evidence that cells with sub-physiological calcium influx are smaller than those with normal calcium gradients and signaling. With hiPSC-CMs in vitro, a unique opportunity is present to track force development over time while modifying extracellular calcium levels directly. Data show increases in hiPSC-CMs total force after being cultured in physiologic calcium levels (1.8 mM) compared to the standard RPMI calcium levels (0.4 mM) employed by several groups, even when assayed in standard 0.4 mM media. All other hiPSC-CMs in this paper were cultured in standard RPMI with standard concentrations.

This is important because calcium signaling is crucial for cardiac myocytes in terms of excitation-contraction coupling[43, 50], as well as signaling through calcium binding proteins, such as calmodulin[51] and calcineurin[52].The increase in Rcan1.4 that we observed in hiPSC-CMs treated with physiological calcium concentrations supports the possibility that the physiologic changes force observed are mechanistically linked to enhanced calcineurin/NFAT signaling. We cannot, however, exclude the potential for activation of other maturation signaling pathways initiated by the two-week culturing of the hiPSC-CMs in physiological extracellular Ca^2+^ conditions. Collectively, this is evidence that differentiation and development protocols that attempt to recapitulate embryonic development should take into consideration the concentration of calcium in the culture media.

Our data provide evidence of an inverse relationship between peak force and substrate stiffness. This should be a significant consideration for ongoing and future studies attempting to translate hiPSC-CMs for regenerative therapies for the diseased myocardium in vivo. Because the stiffness of the human heart increases during development, and fetal myocardium is significantly softer than adult myocardium[26, 27], the ability of an hiPSC-CM to produce force against soft versus stiff substrates may point to the developmental age of the cell. We utilized here three moduli that are in line with the reported range that a cardiac myocyte encounters as the heart develops from an embryonic state to adult[27]. Data show that both total force and normalized force decrease as substrate modulus increases from 3.1 kPa to 9.8 kPa—13.5 kPa, in agreement with a recent report[27]. These findings are in contrast to an earlier study[15]; however, it is difficult to directly compare findings due to the high variability of force values in that earlier work.

The negative correlation between modulus and force development is consistent with the idea that hiPSC-CMs represent an immature state, preferring to contract against a softer substrate. Immature cardiac myocytes, to some degree, lack the cellular machinery to adequately transmit force to their environment[53]. It has also been shown that alterations in the proteins connecting the cytoskeleton to the extracellular makeup affect the traction of cells on stiffer substrates[54], and that immature cardiac myocytes have incompletely developed dystrophin-glycoprotein complexes[25]. Thus, we would expect immature cardiac myocytes to produce force less efficiently against a stiffer substrate, as shown here for hiPSC-CMs.

Finally, we elucidate strain energy applied by hiPSC-CMs to the surrounding environment. Strain energy characterizes the work done by the cell on the underlying substrate, which is fundamental to the force output of contractile cells, including hiPSC-CMs. It has been shown that fibroblasts generate similar amounts of strain energy on substrates of different stiffness[55]. However, as shown here, hiPSC-CMs generate less strain energy in the deformation of stiffer substrates. We posit that hiPSC-CMs, due to their immaturity, are better able to produce force against a substrate that more closely mimics the stiffness of an embryonic heart than that of an adult or fibrotic heart. We speculate that further understanding of strain energy in hiPSC-CMs may help decipher the basis of the difficulties encountered by groups attempting to transplant immature hiPSC-CMs in stiff, diseased adult myocardium[4].

Taken together, these new data show that the functionality of hiPSC-CMs, as determined by their ability to produce force against a substrate via single cell TFM, is a valuable quantitative functional marker that closely resembles that of an immature cardiac myocyte. Specifically, in terms of the impact of geometry on hiPSC-CM contractility, total normalized force production by single myocyte TFM is similar to that of neonatal rat cardiac myocytes. As hiPSC-CMs perform more optimally working on less stiff substrates, we report hiPSC-CMs function is comparable to neonatal cardiac myocytes. In addition, this study shows the important role of length of differentiation in culture and the effects of extracellular calcium concentration in the culture media on the maturation of hiPSC-CMs. Ultimately, quantitative analysis of hiPSC-CM contractile performance as done here via traction force microscopy and using micropatterned platforms with substrates of differing stiffness in the developmental range of heart tissue will be critical toward optimization of culture conditions and cell environment, including matrices, to advance the maturation state of hiPSC-CM toward human adult myocardium.

## Experimental procedures

### Culture and differentiation of human iPSC-derived cardiac myocytes

Human iPSC line DF 19-9-11T, which was derived from healthy donor fibroblasts using a vector-free episomal induction method[56], was graciously provided to us by the laboratory of Dr. Timothy Kamp at the University of Wisconsin-Madison. HiPSCs were cultured according to the protocol outlined in that paper; briefly, cells were grown in TESR-E8 media (Stemcell, Vancouver, CA), on Matrigel-coated (Corning, Corning, NY) 35 mm 6 well plates, and passaged every 4 days via EDTA with a dilution factor of 1:12.

HiPSCs were differentiated according using a small molecule Wnt/GSK3 inhibition protocol^2^. Briefly, hiPSCs were cultured to approximately 90% confluency, then treated with a GSK3 inhibitor, CHIR99021 (Stemgent, Cambridge, MA) in RPMI supplemented with B-27 minus insulin (Thermo Fisher, Waltham, MA) and Matrigel, for 24 hours. Media was replaced for 48 hours. Cells were treated with IWP-4 (Stemgent, Cambridge, MA) in RPMI with HEPES with B-27 minus insulin for 48 hours, and media was replaced every 48 hours until cells began to beat spontaneously, at which point they were grown in RPMI with HEPES supplemented with insulin-replete B-27, with a Ca^2+^ concentration 0.4 mM (standard RPMI concentration). Media was changed every 2–3 days until ready for assays. Higher-calcium (physiological) media was prepared by supplement RPMI + B27 growth media to 1.8 mM Ca^2+^ using 1M CaCl, and then stirred to dissolve any precipitations that form. Cells grown in physiological calcium media had media changed every 1–2 days, as these cells produce acidic media more rapidly. Each condition tested in this paper used data pooled from hiPSC-CMs from 3–4 independent differentiations.

### Isolation and culture of neonatal rat ventricular myocytes

All methods for handling laboratory animals were approved by the Institutional Animal Care and Use Committee at the University of Minnesota. One-day old Sprague Dawley rat pups were sacrificed via decapitation and hearts excised through the chest. Cardiac myocytes were isolated using sequential trypsin and collagenase treatments according to the protocol provided with the Worthington Neonatal Cardiomyocyte Isolation System (Worthington Biochemical, Lakewood, NJ). NRVMs were plated directly onto patterned polyacrylamide constructs at a density of 100,000 cells per well and cultured in DMEM + 4% FBS. Media was changed after 24 hours, and cells were allowed to continue to adhere for 48 more hours until assaying. NRVMs were treated the same way as hiPSC-CMs during the assay procedure.

### Micropatterning of polyacrylamide constructs

Micropatterning stamps were created according to protocols outline by Wang et al[57]. Briefly, photomasks were designed in AutoCAD (AutoDesk, Mill Valley, CA) and printed by Fineline Imagine (Colorado Springs, CO). Single cell shapes were placed far enough apart to ensure contraction of one cell would not affect substrate deformation of neighboring cells. Stamp masters were created using photolithography by applying photomasks to silicon wafers coated with photoresist and exposing to light. Stamps were created by curing polydimethylsiloxane (PDMS) (Dow Corning Sylgard 184, Ellsworth Adhesives) on the patterned silicon master (for patterned stamp) or on an unpatterned silanized silicon wafer (for blank stamps). Both patterned and unpatterned stamps were made new each time they were used. Stamps were coated and stamped according to the stamp-off protocol laid out by Desai et al[58]. Briefly, blank stamps were coated with laminin (50 μg/ml in molecular biology grad water) and incubated for 60 minutes, then dried and inverted onto patterned stamps which have been UV-activated. Blank stamps were immediately peeled off and placed onto 15 mm coverslips which had been plasma-activated by running through a blue flame. Cover slips were then ready for use with gels.

### Polyacrylamide gel construction

Polyacrylamide gels were made using ratios of Acrylamide to N,N’-methylenebisacrylamide according to Tse et al[59] and then actual gel stiffness was measured using uniaxial stress testing (S1 Table). Ahead of time, 25 mm glass coverslips were UV treated, then treated with 3-aminopropyltriethoxysilane for 3 minutes, and rinsed with ethanol. Polyacrylamide was made with 1x phosphate-buffered saline, and 0.2 μm red FluoSpheres fluorescent beads (Thermo Fisher, Waltham, MA) were mixed into the unpolymerized acrylamide solution at a concentration of 0.005% (diluted 1:200). The solution was degassed for 15 minutes, and to the unpolymerized solution was added tetramethylethylenediamine (final dilution 1:1500), and the solution was brought to a pH of 7 via HCl. Ammonium persulfate (final concentration 0.017% w/v) and N-hydroxysuccinimide ester (final concentration 0.0083 mg/ml) were added to solution. 15 μl of solution were quickly pipetted onto APS-treated coverslips. Laminin-coated coverslips were inverted onto the solution and allowed to polymerize at room temperature for 60 minutes. Top coverslips were removed from the polymerized gels, and gels were incubated in 4% BSA at 37° for 45 minutes, then rinsed 3 times with 1x PBS.

HiPSCs were dissociated in Accutase (Thermo Fisher, Waltham, MA) for 20 minutes, then resuspended in warm RPMI + B27 and replated onto polyacrylamide gels at 100,000 cells per well. Cells adhered overnight, then media was changed the following morning. Cells were allowed to adhere for two more days, then assayed on day 3 after plating.

### Traction force microscopy and analysis

Experiments were performed on an Olympus X81 Inverted Microscope using a 40x UPLSAPO40X2, NA 0.95 objective in an environmental control chamber at 37°C. Images were acquired using MetaMorph software (Molecular Devices, Sunnyvale, CA) at a rate of 3.5 frames per second. Cells were paced at 0.5 Hz with a 35 mV square pulse using a MyoPacer field stimulator (IonOptix, Westwood, MA) in RPMI + B27 with HEPES. Cells were paced for 3–5 minutes before recording, and data was recorded from each dish for no more than 30 minutes to avoid recording from dying cells. Cells were given fresh media 60 minutes before data acquisition.

Images were analyzed using ImageJ code developed by Tseng et al[60, 61]. Stacks of images of the cell and fluorescent beads before and during contraction were oriented vertically and cropped to an area of 64.4 μm wide by 128.8 μm tall before analysis. Particle image velocimetry using iterative interrogation windows of 128-64-32 pixel width was completed between the matched bead images from the same cell location at different time points. The noise-filtered displacement field was used to calculate traction stresses with a Fourier transform traction cytometry (FTTC) ImageJ plugin using the Fourier transform traction cytometry method[51]. A regularization factor of 1x10-9 was applied for improved accuracy, as described in Stricker et al[50]. Stress vector magnitudes were integrated over the entire 64.4 μm by 128.8 μm area of interest and reported as total force in μN. Normalized force was calculated by dividing total force by the area of the cell and is reported in μN/μm^2^ x 10^−5^. Strain energy of the substrate was calculated for each individual hiPSC-CM as[48]:

$$U=\frac{1}{2}\int T∙udxdy$$

where **T** is the traction stress and **u** is the displacement.

### Real-time PCR

Rcan1.4 primers were synthesized by Integrative DNA Technology (Coralville, IA) using the primer sequences shown below. RNA was extracted from dissociated hiPSC-CMs using the RNEasy Plus kit (Qiagen, Hilden, Germany) and quantified by NanoDrop spectrophotometry (NanoDrop, Wilmington, DE). Next, cDNA libraries were generated using the Superscript Vilo kit (Thermo Fisher, Waltham, MA) and quantitative real-time PCR was performed using the Bio-Rad SYBR Green qPCR Master Mix Bio-Rad, Hercules, CA) on an Eppendorf Mastercycler machine (Eppendorf, Hamburg, Germany). Analysis of expression and fold change was carried out using the ddCt method[62], with GAPDH used as a housekeeping gene.

**F**: AGAGGTACCACAGGAAGCC

**R**: ATGAGGGAAGAAAGGAAACGC

### Statistical methods

All statistical analysis was performed using Prism software (GraphPad, San Diego, CA). Correlation analysis was done by linear regression, and estimation of significant non-zero slopes was determined. Significant differences between groups for all other experiments was estimated by one-way ANOVA with a Tukey’s post hoc test. Significant differences in Fig 5 were estimated with a Student’s T test. Spline plots in Fig 1 were created using Matlab (Mathworks, Natick, MA).

## Supporting information

S1 Movie HiPSC-CM displacement of fluorescent beads in the hydrogel substrate. (AVI)

S1 Table Measurements of hydrogel stiffness. (TIF)

## References

[1] Paige SL, Osugi T, Afanasiev OK, Pabon L, Reinecke H, Murry CE. Endogenous Wnt/beta-catenin signaling is required for cardiac differentiation in human embryonic stem cells. PLoS One. 2010; 5: e11134 doi: 10.1371/journal.pone.0011134 20559569 10.1371/journal.pone.0011134 PMC2886114

[2] Lian X, Hsiao C, Wilson G, Zhu K, Hazeltine LB, Azarin SM et al Robust cardiomyocyte differentiation from human pluripotent stem cells via temporal modulation of canonical Wnt signaling. Proc Nat Acad Sci USA. 2012; 109: 1848–1857. 10.1073/pnas.1200250109 PMC3390875 22645348

[3] Zhang J, Klos M, Wilson GF, Herman AM, Lian X, Raval KK et al Extracellular matrix promotes highly efficient cardiac differentiation of human pluripotent stem cells: the matrix sandwich method. Circ Res. 2012; 111: 1125–1136. doi: 10.1161/CIRCRESAHA.112.273144 22912385 10.1161/CIRCRESAHA.112.273144 PMC3482164

[4] Laflamme MA, Chen KY, Naumova AV, Muskheli V, Fugate JA, Dupras SK et al Cardiomyocytes derived from human embryonic stem cells in pro-survival factors enhance function of infarcted rat hearts. Nat Biotechnol. 2017; 25: 1015–1024. 10.1038/nbt1327 17721512

[5] Shimizu T, Shiohara M, Tai T, Nagao K, Nakajima K, Kobayashi H. Derivation of integration-free iPSCs from a Klinefelter syndrome patient. Reprod Med Biol. 2016; 15: 35–43. doi: 10.1007/s12522-015-0213-9 26709348 10.1007/s12522-015-0213-9 PMC4686545

[6] Hashimoto A, Naito AT, Lee JK, Kitazume-Taneike R, Ito M, Yamaguchi T et al Generation of induced pluripotent stem cells from patients with Duchenne muscular dystrophy and their induction to cardiomyocytes. Int Heart J. 2016; 57: 112–117. doi: 10.1536/ihj.15-376 26673445 10.1536/ihj.15-376

[7] Ivaschenko CY, Pipes GC, Lozinskaya IM, Lin Z, Xiaoping X, Needle S et al Human induced pluripotent stem cell-derived cardiomyocytes exhibit temporal changes in phenotype. Am J Physiol Heart Circ Physiol. 2013; 305: 913–922. 10.1152/ajpheart.00819.2012 23832699

[8] Kuzmenkin A, Lian H, Xu G, Pfannkuche K, Eichhorn H, Fatima A et al Functional characterization of cardiomyocytes derived from murine induced pluripotent stem cells in vitro. FASEB J. 2009; 23: 4168–4180. doi: 10.1096/fj.08-128546 19703934 10.1096/fj.08-128546

[9] Bedada FB, Chan SS, Metzger SK, Zhang L, Zhang J, Garry DJ et al Acquisition of a quantitative, stoichiometrically conserved ratiometric marker of maturation status in stem cell-derived cardiac myocytes. Stem Cell Reports. 2014; 3: 594–605. doi: 10.1016/j.stemcr.2014.07.012 25358788 10.1016/j.stemcr.2014.07.012 PMC4223713

[10] Ribeiro MC, Tertoolen LG, Guadix JA, Bellin M, Kosmidis G, D’Aniello C et al Functional maturation of human stem cell-derived cardiomyocytes in vitro- correlation between traction force and electrophysiology. Biomaterials. 2015; 51: 138–150. doi: 10.1016/j.biomaterials.2015.01.067 25771005 10.1016/j.biomaterials.2015.01.067

[11] Heubsch N, Loskill P, Devashwar N, Spencer CI, Judge LM, Mandegar MA et al Miniaturized iPS-cell-derived cardiac muscles for physiologically relevant drug response analyses. Sci Rep. 2016; 6: 24726 doi: 10.1038/srep24726 27095412 10.1038/srep24726 PMC4837370

[12] Pfannkuche K, Liang H, Xi J, Fatima A, Nguemo F, Matzkies M et al Cardiac myocytes derived from murine reprogrammed fibroblasts: intact hormonal regulation, cardiac ion channel expression and development of contractility. Cell Physiol Biochem. 2009; 24: 73–86. doi: 10.1159/000227815 19590195 10.1159/000227815

[13] Sheehy SP, Pasqualini F, Grosberg A, Park SJ, Aratyn-Schaus Y, Parker KK. Quality metrics for stem cell-derived cardiac myocytes. Stem Cell Reports. 2014; 2: 282–294. doi: 10.1016/j.stemcr.2014.01.015 24672752 10.1016/j.stemcr.2014.01.015 PMC3964283

[14] Buessman KM, Rodriguez ML, Leonard A, Taparia N, Thompson CR. Sniadecki NJ. Micropost arrays for measuring stem cell-derived cardiomyocyte contractility. Methods. 2016; 94: 43–50. doi: 10.1016/j.ymeth.2015.09.005 26344757 10.1016/j.ymeth.2015.09.005 PMC4761463

[15] Hazeltine LB, Simmons CS, Salick MR, Lian X, Badur MG, Han W et al Effects of substrate mechanics on contractility of cardiomyocytes derived from human pluripotent stem cells. Int J Cell Biol. 2012; 2012: 508294 doi: 10.1155/2012/508294 22649451 10.1155/2012/508294 PMC3357596

[16] Pioner JM, Racca AW, Klaiman JM, Yang KC, Guan X, Pabon L et al Isolation and mechanical measurements of myofibrils from human induced pluripotent stem cell derived cardiomyocytes. Stem Cell Reports. 2016; 6: 885–896. doi: 10.1016/j.stemcr.2016.04.006 27161364 10.1016/j.stemcr.2016.04.006 PMC4911495

[17] Seidner S, Kruger M, Schroeter M, Metzler D, Roell W, Fleischmann BK et al Developmental changes in contractility and sarcomeric proteins from the early embryonic to the adult stage in the mouse heart. Physiol. 2003; 548: 493–505. 10.1113/jphysiol.2002.036509 PMC2342849 12640016

[18] Posterino GS, Dunn SL, Botting KJ, Wang W, Gentili S, Morrison JL. Changes in cardiac troponins with gestational age explain changes in cardiac muscle contractility in the sheep fetus. J Appl Physiol. 2011; 111: 236–245. doi: 10.1152/japplphysiol.00067.2011 21493721 10.1152/japplphysiol.00067.2011

[19] Jones HJ, Keep RF. The control of potassium concentration in the cerebrospinal fluid and brain interstitial fluid of developing rats. J Physiol. 1987; 383: 441–453. 3656129 10.1113/jphysiol.1987.sp016419 PMC1183080

[20] Cannell MB, Cheng H, Lederer WJ. The control of calcium release in heart muscle. Science. 1995; 268: 1045–1049. 7754384 10.1126/science.7754384

[21] Bers DM. Cardiac excitation-contraction coupling. Nature. 2002; 415: 198–205. doi: 10.1038/415198a 11805843 10.1038/415198a

[22] Moore GE, Gerner RE, Franklin HA. Culture of normal human leukocytes. JAMA. 1967l 199: 519–524. 4960081

[23] Li M, Wang N, Gong HQ, Li WZ, Liao XH, Yang XL et al Ca2+ signal-induced cardiomyocyte hypertrophy through activation of myocardin. Gene. 2015; 557: 43–51. doi: 10.1016/j.gene.2014.12.007 25485719 10.1016/j.gene.2014.12.007

[24] Andersen ND, Ramachandran KV, Bao MM, Kirby ML, Pitt GS, Hutson MR. Calcium signaling regulates ventricular hypertrophy through development independent of contraction or blood flow. J Mol Cell Cardiol. 2015; 80: 1–9. doi: 10.1016/j.yjmcc.2014.12.016 25536179 10.1016/j.yjmcc.2014.12.016 PMC4346462

[25] Kuo PL, Lee H, Bray MA., Geisse NA, Huang YT, Adams WJ et al Myocyte shape regulates lateral registry of sarcomeres and contractility. Am J Pathol. 2012; 181: 2030–2017. doi: 10.1016/j.ajpath.2012.08.045 23159216 10.1016/j.ajpath.2012.08.045 PMC3509763

[26] Bray MA, Sheehy SP, Parker KK. Sarcomere alignment is regulated by myocyte shape. Cell Motil Cytoskeleton. 2008; 65: 641–651. doi: 10.1002/cm.20290 18561184 10.1002/cm.20290 PMC4492320

[27] Ribeiro AJ, Ang YS, Fu JD, Rivas RN, Mohamed TM, Higgs GC et al Contractility of single cardiomyocytes differentiated from pluripotent stem cells depends on physiological shape and substrate stiffness. Proc Nat Acad Sci USA. 2015; 112: 12705–12710. doi: 10.1073/pnas.1508073112 26417073 10.1073/pnas.1508073112 PMC4611612

[28] Foglia MJ, Poss KD. Building and re-building the heart by cardiomyocyte proliferation. Development. 2016; 143: 729–740. doi: 10.1242/dev.132910 26932668 10.1242/dev.132910 PMC4813344

[29] Hersch N, Wolters B, Dreissen G, Springer R, Kirchgessner N, Merkel R et al The constant beat: cardiomyocytes adapt their force by equal contraction upon environmental stiffening. Biol Open. 2012; 2: 351–361. 10.1242/bio.20133830 PMC3603417 23519595

[30] ter Keurs HE. Heart failure and Starling’s law of the heart. Can J Cardiol. 1996; 12: 1047–1057. 9191498

[31] Mattei G, Ahluwalia A. Sample, testing and analysis variables affecting liver mechanical properties: A review. Acta Biomater. 2016; 45: 60–71. doi: 10.1016/j.actbio.2016.08.055 27596489 10.1016/j.actbio.2016.08.055

[32] Jacot J.G, Martin JC, Hunt DL. Mechanobiology of cardiomyocyte development. J Biomech. 2010; 43: 93–98. doi: 10.1016/j.jbiomech.2009.09.014 19819458 10.1016/j.jbiomech.2009.09.014 PMC2813357

[33] Chang WT, Chen JS, Tsai MH, Juang JN, Liu PY. Interplay of aging and hypertension in cardiac remodeling: a mathematical geometric model. PLoS One. 2016; 11: e0168071 doi: 10.1371/journal.pone.0168071 27977729 10.1371/journal.pone.0168071 PMC5158006

[34] Boothe SD, Meyers JD, Pok S, Sun J, Xi Y, Nieto RM et al The effect of substrate stiffness on cardiomyocyte action potentials. Cell Biochem Biophys. 2016; 74: 527–535. doi: 10.1007/s12013-016-0758-1 27722948 10.1007/s12013-016-0758-1 PMC5102789

[35] Young JL, Kretchmer K, Ondeck MG, Zambon AC, Engler AJ. Mechanosensitive kinases regulate stiffness-induced cardiomyocyte maturation. Sci Rep. 2014; 19: 6425. 10.1038/srep06425 PMC4168277 25236849

[36] Echegaray K, Andreu I, Lazkano A, Villanueva I, Saenz A, Elizalde MR et al Role of myocardial collagen in severe aortic stenosis with preserved ejection fraction and symptoms of heart failure. Rev Esp Cardiol. 2017; 16: 30462–30465. 10.1016/j.rec.2016.12.038 28215921

[37] Davis J, Wen H, Edwards T, Metzger JM. Thin filament disinhibition by restrictive cardiomyopathy mutant R193H troponin I induces Ca2+-independent mechanical tone and acute myocyte remodeling. Circ Res. 2007; 100: 1494–1502. doi: 10.1161/01.RES.0000268412.34364.50 17463320 10.1161/01.RES.0000268412.34364.50

[38] Lopez-Redondo F, Kurokawa J, Nomura F, Kaneko T, Hamada T, Furukawa T et al A distribution analysis of action potential parameters obtained from patch-clamped human stem cell-derived cardiomyocytes. J Pharmacol Sci. 2016; 131: 141–145. doi: 10.1016/j.jphs.2016.04.015 27179939 10.1016/j.jphs.2016.04.015

[39] Jones AR, Edwards DH, Cummins MJ, Williams AJ, George CH. A systematized approach to investigate Ca2+ synchronization in clusters of human induced pluripotent stem cell-derived cardiomyocytes. Front Cell Dev Biol. 2016; 3: 89 doi: 10.3389/fcell.2015.00089 26793710 10.3389/fcell.2015.00089 PMC4710702

[40] Boheler KR, Joodi RN, Qiao H, Juhasz O, Urick AL, Chuppa SL et al Embryonic stem cell-derived cardiomyocyte heterogeneity and the isolation of immature and committed cells for cardiac remodeling and regeneration. Stem Cells Int. 2011; 2011: 214203 doi: 10.4061/2011/214203 21912557 10.4061/2011/214203 PMC3168772

[41] Robertson C, Tran DD, George SC. Concise review: maturation phases of human pluripotent stem cell-derived cardiomyocytes. Stem Cells. 2013; 31: 829–837. doi: 10.1002/stem.1331 23355363 10.1002/stem.1331 PMC3749929

[42] Tham YK, Bernardo BC, Ooi JY, Weeks KL, McMullen JR. Pathophysiology of cardiac hypertrophy and heart failure: signaling pathways and novel therapeutic targets. Arch Toxicol. 2015; 89: 1401–1438. doi: 10.1007/s00204-015-1477-x 25708889 10.1007/s00204-015-1477-x

[43] Pelham Jr. RJ, Wang YI. Cell locomotion and focal adhesions are regulated by substrate flexibility. Proc Nat Acad Sci USA. 1997; 94: 13661–13665. 9391082 10.1073/pnas.94.25.13661 PMC28362

[44] Benninger C, Kadis J, Prince DA. Extracellular calcium and potassium changes in hippocampal slices. Brain Res. 1980; 187: 165–182. 7357467 10.1016/0006-8993(80)90502-8

[45] Miller DJ. Sydney Ringer; physiological saline, calcium, and the contraction of the heart. J Physiol. 2004; 555: 585–587. doi: 10.1113/jphysiol.2004.060731 14742734 10.1113/jphysiol.2004.060731 PMC1664856

[46] Tyrode MV. The mode of action of some purgative salts. Arch Int Pharmacodyn Ther. 2010; 17: 205–209.

[47] Yang J, Rothermel B, Vega RB, Frey N, McKinsey TA, Olsen EN et al Independent signals control expression of the calcineurin inhibitory proteins MCIP1 and MCIP2 in striated muscles. Circ Res. 2000; 87: E61–E68. 11110780 10.1161/01.res.87.12.e61

[48] Martiel JL, Leal A, Kurzawa L, Balland M, Wang I, Vignaud T et al Measurement of cell traction forces with ImageJ. Methods Cell Biol. 2015; 125: 269–287. doi: 10.1016/bs.mcb.2014.10.008 25640434 10.1016/bs.mcb.2014.10.008

[49] Stricker J, Sabass B, Schwarz US, Gardel ML. Optimization of traction force microscopy for micron-sized focal adhesions. J Phys Condens Matter. 2010; 22: 194104 doi: 10.1088/0953-8984/22/19/194104 20523913 10.1088/0953-8984/22/19/194104 PMC2879600

[50] Elustondo PA, Nichols M, Robertson GS, Pavlov EV. Mitochondrial Ca2+ uptake pathways. J Bioenerg Biomembr. 2017; 49: 113–119. doi: 10.1007/s10863-016-9676-6 27665468 10.1007/s10863-016-9676-6

[51] Sorensen AB, Sondergaard MT, Overgaard MT. Calmodulin in a heartbeat. FEBS J. 2013; 280: 5511–532. doi: 10.1111/febs.12337 23663249 10.1111/febs.12337

[52] Wang Y, Tandan S, Hill JA. Calcineurin-dependent ion channel regulation in heart. Trends Cardiovasc Med. 2014; 24: 14–22. doi: 10.1016/j.tcm.2013.05.004 23809405 10.1016/j.tcm.2013.05.004 PMC3830706

[53] Raeker MO, Shavit JA, Dowling JJ, Michele DE, Russell MW. Membrane-myofibril crosstalk in myofibrillogenesis and in muscular dystrophy pathogenesis: lessons from the zebrafish. Front In Phys. 2014; 5: 14. 10.3389/fphys.2014.00014 PMC3904128 24478725

[54] Elosegui-Artola A, Oria R, Chen Y, Kosmalska A, Perez-Gonzalez C, Castro N et al Mechanical regulation of a molecular clutch defines force transmission and transduction in response to matrix rigidity. Nat Cell Bio. 2016; 18: 549–548. 27065098 10.1038/ncb3336

[55] Oakes PW, Banerjee S, Marchetti M, Gardel ML. Geometry regulates traction stresses in adherent cells. Biophys J. 2014; 107: 825–833. doi: 10.1016/j.bpj.2014.06.045 25140417 10.1016/j.bpj.2014.06.045 PMC4142236

[56] Yu J, Hu K, Smuga-Otto K, Tian S, Stewart R, Slukvin II et al Human induced pluripotent stem cells free of vector and transgene sequences. Science. 2009; 324: 797–801. doi: 10.1126/science.1172482 19325077 10.1126/science.1172482 PMC2758053

[57] Wang N, Ostuni E, Whitesides GM, Ingber DE. Micropatterning tractional forces in living cells. Cell Motil Cytoskeleton. 2002; 52: 97–106. doi: 10.1002/cm.10037 12112152 10.1002/cm.10037

[58] Desai RA, Rodriguez NM, Chen CS. “Stamp-off” to micropattern sparse, multicomponent features. Methods Cell Biol. 2004; 119: 3–16. 10.1016/B978-0-12-416742-1.00001-9 24439276

[59] Tse JR, Engler AJ. Preparation of hydrogel substrates with tunable elastic properties. Curr. Protoc. Cell Biol. 2010; 10: 16 doi: 10.1002/0471143030.cb1016s47 20521229 10.1002/0471143030.cb1016s47

[60] Tseng Q, Duchemin-Pelletier E, Deshiere A, Balland M, Guillou H, Filhol O et al Spatial organization of the extracellular matrix regulates cell-cell junction positioning. Proc Nat Acad Sci USA 2012; 109: 1506–1511. doi: 10.1073/pnas.1106377109 22307605 10.1073/pnas.1106377109 PMC3277177

[61] Sabass B, Gardel ML, Waterman CM, Schwarz US. High resolution traction force microscopy based on experimental and computational advances. Biophys J. 2008; 94: 207–220. doi: 10.1529/biophysj.107.113670 17827246 10.1529/biophysj.107.113670 PMC2134850

[62] Livak KJ and Schmittgen TD. Analysis of relative gene expression data using real-time quantitative PCR and the 2(-Delta Delta C(T)) Method. Methods. 2001; 25: 402–408. doi: 10.1006/meth.2001.1262 11846609 10.1006/meth.2001.1262

