# Supplementary figures and images for: Investigation of human iPSC-derived cardiac myocyte functional maturation by single cell traction force microscopy
Source: PLoS One. 2018 Apr 4;13(4):e0194909. doi: 10.1371/journal.pone.0194909 (PMC5884520; doi:10.1371/journal.pone.0194909)

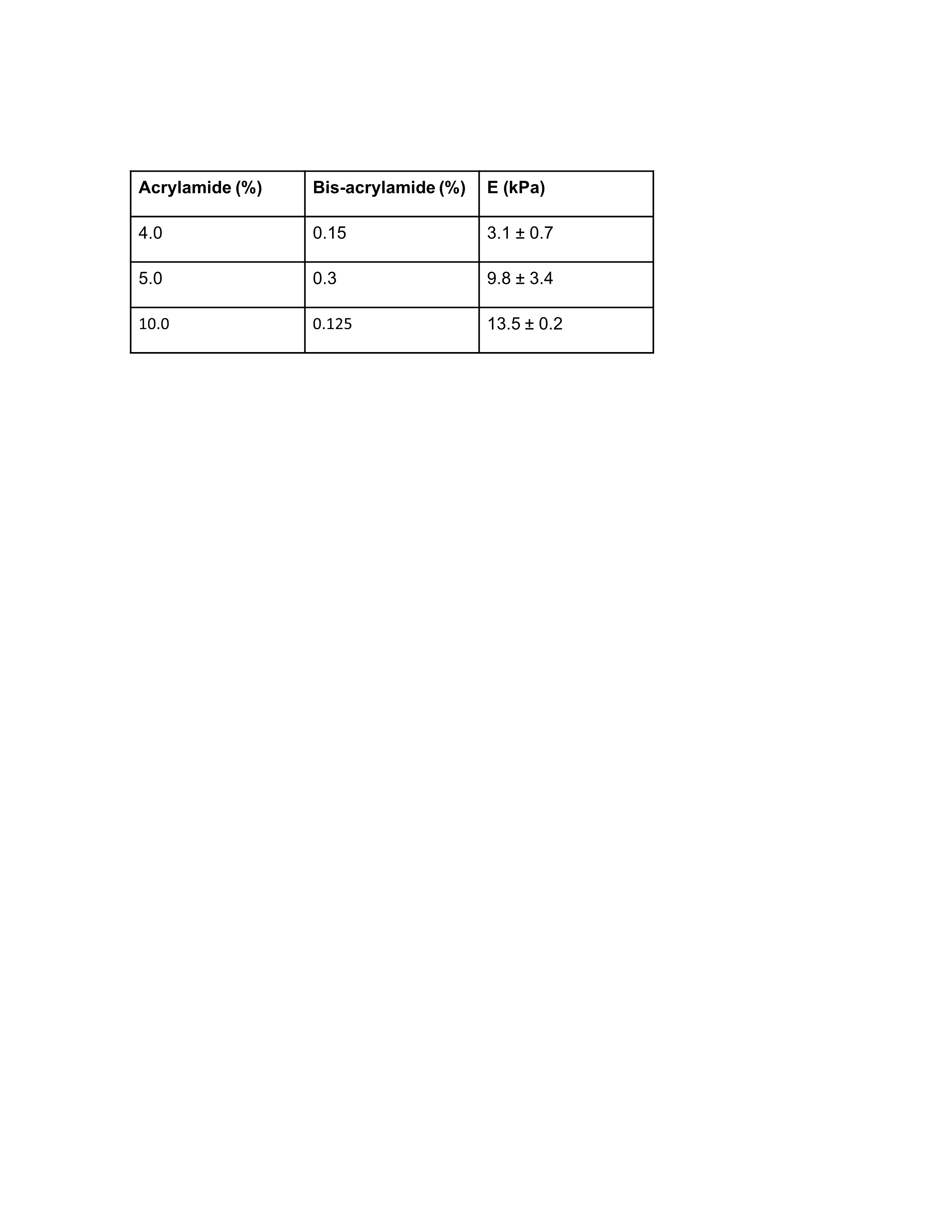

Supplement: S1 Table — (TIF) [file pone.0194909.s002.tif]
